# Effect of COVID-19 pandemic on medical waste management: a case study

**DOI:** 10.1007/s40201-021-00650-9

**Published:** 2021-03-18

**Authors:** Roshanak Rezaei Kalantary, Arsalan Jamshidi, Mohammad Mehdi Golbini Mofrad, Ahmad Jonidi Jafari, Neda Heidari, Saeid Fallahizadeh, Mohsen Hesami Arani, Javad Torkashvand

**Affiliations:** 1 Research Center for Environmental Health Technology, Iran University of Medical Sciences, Tehran, Iran; 2 Department of Environmental Health Engineering, School of Public Health, Iran University of Medical Sciences, Tehran, IR Iran; 3 Department of Environmental Health Engineering, School of Health and Nutrition Sciences, Yasuj University of Medical Sciences, Yasuj, Iran; 4 IHE Delft Institute for Water Education, Delft, The Netherlands; 5 Environment Research Center, Research Institute for Primordial Prevention of Non-communicable Disease, Department of Environmental Health Engineering, School of Health, Isfahan University of Medical Sciences, Isfahan, Iran

**Keywords:** Medical waste, COVID-19, Waste composition, Iran

## Abstract

Covid-19 Pandemic leads to medical services for the society all over the world. The Covid-19 pandemic influence the waste management and specially medical waste management. In this study, the effect of the Covid-19 outbreak on medical waste was evaluated via assessing the solid waste generation, composition, and management status in five hospitals in Iran. The results indicated that the epidemic Covid-19 leads to increased waste generation on average 102.2 % in both private and public hospitals. In addition, the ratio of infectious waste in the studied hospitals increased by an average of 9 % in medical waste composition and 121 % compared with before COVID-19 pandemic. Changes in plans and management measurement such as increasing the frequency of waste collection per week leads to lower the risk of infection transmission from medical waste in the studied hospitals. The results obtained from the present research clearly show the changes in medical waste generation and waste composition within pandemic Covid-19. In addition, established new ward, Covid-19 ward with high-infected waste led to new challenges which should be managed properly by change in routine activities.

## Introduction

Ever increasing population growth in cities in pace with economic growth has led to an increased production of various types of municipal solid waste, in particular, medical waste [1]. Proper management of municipal waste in order to reduce its adverse effects on the environment and the health of citizens is a necessity [2–4]. Different types of municipal solid waste have numerous effects on health, environmental, economic and social aspects; Nowadays, the littered waste management is considered in priority [5]. Amongst the types of municipal solid waste, medical waste is important due to its potential infection increased generation rate [1]. Medical waste, depending on their sources includes hospital waste, dental waste, medical laboratory waste and etc.[6–8]. Hospital waste is one of the most important medical wastes, which include different types of infectious, sharp, toxic, chemical and pharmaceutical, and semi-household wastes [9, 10]. Proper management of medical waste in order to control its corresponding risks on health and to prevent the transmission of infectious such as hepatitis, AIDS, and typhoid is a necessity. The medical waste management elements include waste segregation, storage, transportation, disinfection and final disposal [1]. Pandemic Covid-19 leads to increases in patient and healthcare activities; one of the consequences of Covid-19 is its effect on the quantity and composition of medical waste [11, 12]. The Covid-19 pandemic with change the influence the lifestyle influence the quantity and composition of municipal solid waste; waste management in this condition is done according to new guidelines [13]. Covid-19 pandemic has increased the waste generation and the proportion of infectious waste in the landfill; one of the concerns raised in this area is the littering of masks and gloves by citizens which are potentially infectious [13]. Furthermore, caring for sick or suspected people at home has led to the production of infectious waste in addition to hospitals waste. The aim of this study was to evaluate the effects of Covid-19 on the quantity, composition and management of medical waste in Iran in order to identify the challenges posed by the epidemic on medical waste management. To this end, five hospitals in different cities were surveyed and their waste management elements activities were compared with before Covid-19 pandemic.

## Method

In this study, five hospitals including two private hospitals and three public hospitals in different cities of Iran were surveyed in terms of waste generation, waste composition and waste management. The general characteristics of waste management activities for the studied hospitals is summarized in Table 1. The data were collected by interviews with staff responsible for waste management in different hospitals. This information was provided and collected based on the physical analysis of hospital wastes. The classification of produced waste in studied hospitals was done according to the model presented in Table 2, which is common in medical waste management in Iran [1]. The medical waste composition in the studied hospitals were classified into five categories. In addition, to understand the impact of the epidemic on waste generation, waste composition and the status of hospital waste management, data were reviewed and analyzed in the two period of before and during Covid-19 pandemic via statistical T-Test with 95 % confidence interval.

**Table 1:** General characteristics of the studied hospitals

Hospital	Type	Has Covid ward?	Has other ward?	Number of bed in Covid ward	Number of bed in other ward	With temporary storage?	Disinfection method
A	Private	Yes	Yes	27	156	Yes	Autoclave
B	Public	Yes	Yes	106	91	Yes	Autoclave
C	Private	Yes	Yes	20	44	Yes	Autoclave
D	Public	Yes	No	200	0	Yes	Autoclave
E	Public	Yes	Yes	25	86	Yes	Incinerator

**Table 2:** Category of medical wastes in this study

Type of medical waste	Example
Semi-household waste	Dry paper towel, dry gauze, nylon, plastic, syringe and needle packaging, film packet plastic, mixed gypsum and gauze, paper banderole, food waste, food waste packaging, tea slag, filter tip, mixed soil and gypsum, medicine ampoule packaging
Infectious waste	Blood-contaminated paper towel, blood-contaminated gauze, nylon gloves, latex gloves, syringes, personal protective equipment like mask and gown in Covid-19 pandemic.
Chemical and pharmaceutical waste	Used medicine ampoule, crylic, calcium hydroxide
Sharp waste	Needles, surgical blades
Pathological waste	Tissues

## Results and discussion

The results showed that Covid-19 pandemic has increased the waste generation rate in the studied hospitals. As can be seen in Table 3, the waste generation rate in studied hospitals were not the same; on average, 102.2 % increases were observed for daily waste generation rate in five studied hospitals. The detailed information on waste generation rate in new established ward, Covid-19 ward is summarized in Table 4.

**Table 3:** Medical waste generation in studied hospitals

Hospital	Total waste generation Kgr/day		Infectious waste generation Kgr/day		Total waste generation Kgr/day/bed		Increase ratio in covid-19 compare with before covid-19 %
	Before Covid-19	In Covid-19	Before Covid-19	In Covid-19 (increase)	Before Covid-19	In Covid-19	
A	477	628	267	385 (44.2 %)	2.6	3.43	32
B	370	1060	170	260 (52.9 %)	1.87	5.38	186
C	80	205	35	150 (328.5 %)	1.25	3.2	147
D	370	560	230	500 (117.4 %)	1.85	2.8	52
E	145	280	80	130 (62.5 %)	1.3	2.52	94

**Table 4:** Solid waste generation in some wards of hospital A (kgr/day)

Waste type		ICU	NICU	CCU	Radiography	Post CCU	Emergency
Infectious	Before Covid-19	18	4	7	6	5	28
	In Covid-19	21	6	8	16	10	32
Other	Before Covid-19	6	3	4	4	3	8
	In Covid-19	8	3	5	7	4	11

The increased waste generation rate in healthcare centers during Covid-19 pandemic were reported in similar studies [14]. Evidence shows that during an epidemic, as the experience of Covid-19 pandemic emphasize in this period, the municipal solid waste production will change in the future. However, the changes in waste generation have not been same in the different communities [13]. However, increased medical and hospital waste generation rates in all communities is predictable for some reasons including the increase in the number of patients admitted to hospitals. The results of this study showed that although an increase in waste generation was seen in all hospitals, however, the ratio of increase ranged from 0.82 to 3.5 kg/bed/day. In addition, the results showed the average waste generation rates per patient bed in the time before the epidemic and during the epidemic, were found to be 1.77 and 3.46 kg waste per day per bed, respectively.

**Fig. 1:**
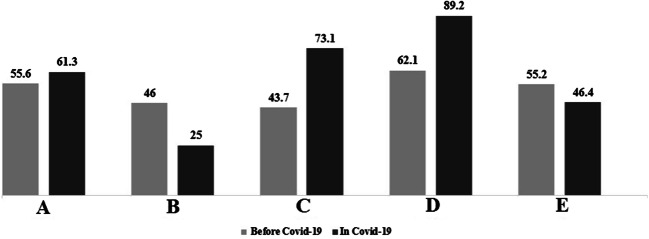
Comparison of infectious waste ratio (%) with before Covid-19 pandemic in the studied hospitals

Another noteworthy point about the results of this study is the increases in the amount of infectious waste in all studied hospitals during the epidemic Covid-19. Given to the increases in total waste production during the epidemic, the ratio of infectious waste in the waste mass has increased in most hospitals; this ratio increased by an average of 9.7 % for the studied hospitals (See Fig. 1).

However, the increases in the use of personal protective equipment by citizens during the epidemic causes the production of waste with the potential infections from the municipal waste [12, 14]; the continuous use of this equipment by the employees and clients, as well as an increase in patients in hospitals leads to increased ratio of infectious wastes in the Covid-19 pandemic. In this condition, hospital waste management will be of particular importance because there is a concern about the possibility of transmitting the disease agent from the waste mass to staff and the community [11, 15].

Considering the increases in waste generation in the studied hospitals and also the increase in the ratio of infectious waste compared to before the Covid-19 pandemic, a change in the hospital waste management activities based the new conditions is necessary to reduce the possibility of disease transmission from the waste mass [13]. The results showed that the management of medical waste in the studied hospitals in the Covid-19 pandemic has a satisfying status, which is due to the proper management of hospital waste as a serious risk to health and the environment in recent decades in Iran [1]. However, in some countries there is concern about the spread of the virus due to poor management of infectious waste [16]. As shown in Table 5, the management of medical waste in the studied hospitals in the stages of segregation, storage, disinfection and transportation shows that the increase in the quantity and infectious potential of waste in Covid-19 pandemic was on range of storage and disinfection equipment in hospitals. The use of autoclave as a common method of waste disinfection in hospitals as well as on-site use of this equipment provides a good model for medical waste management in epidemic. This method of disinfection will reduce the possibility of transmitting the disease agent during the transportation of waste to the final disposal centers. However, an important point in the management of infectious waste due to concerns about the possibility of virus transmission is the reduction of storage time, as reported in South Korea reducing this period from 7 days to less than one day [17]. In all hospitals, due to the appropriate capacity of disinfection equipment, the time of waste storage before disinfection was less than one day, while the collection period of disinfected waste in some hospitals was done after 2–3 days.

**Table 5:** Medical waste activities in Covid-19 pandemic in studied hospitals

Hospital	Waste segregation	Storage before disinfection (day)	Collection and transport after disinfection (in week)	Disinfection method	Use of personal protective equipment	Final disposal method
A	Yes	< 1	7	Autoclave	Yes	Landfill
B	Yes	< 1	7	Autoclave	Yes	Landfill
C	Yes	< 1	6	Autoclave	Yes	Landfill
D	Yes	< 1	2–3	Autoclave	Yes	Landfill
E	Yes	< 1	7	Incineration	Yes	Landfill^*^

* For residue

**Fig. 2:**
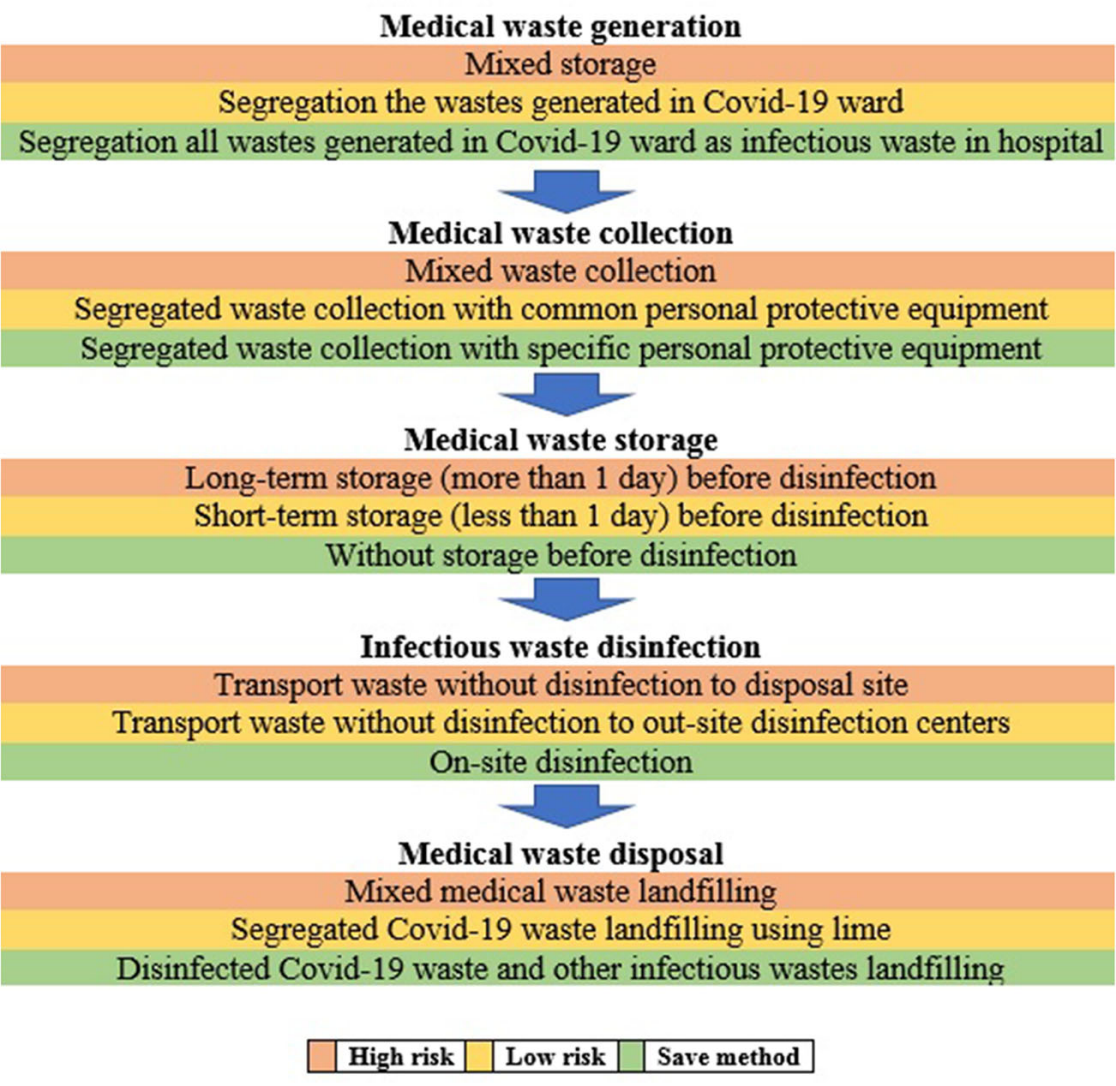
Different plan for medical waste management in epidemic condition

An important point in medical waste management is the segregation of infectious waste from the other waste, especially during the pandemic period [1]. In the studied hospitals, by separating Covid-19 ward from other wards, the waste produced in this ward was generally considered as infectious waste and entered the disinfection stages. However, according to the previous procedure in other words, infectious, sharp and pathological wastes were stored and managed separately. Although these conditions can be effective in reducing the risk of transmission of the virus to medical waste mass and finally to the environment, there are some reports that in Covid-19 pandemic all generated waste in the hospital is considered as infectious waste [18]. However, such precautionary plan will depend on the financial situation and available equipment. However, due to the contamination potential of face masks and gloves used by staff, patients and clients [13], it is necessary to consider special containers to dispose of them separately from the common waste in the hospital. However, this procedure was not observed in the studied hospitals. Based on the classification presented in Fig. 2, taking into account the results of this study and also the use of personal protective equipment by all staff related to hospital waste management and the use of special trucks for waste transport, the status of medical waste management in studied hospitals were evaluated to be in low risk and save status.

## Conclusion

Medical waste generation, composition and management in five hospitals in Covid-19 pandemic in Iran was investigated. One of the effects of Covid-19 pandemic on medical waste was to increase the generation of this type of waste, which in the studied hospitals were found to be 0.95 to 3.51 kg/bed/day. In addition, an increase in the ratio of infectious waste in the medical waste mass was observed by 9 % due to the increase in patients in the infectious ward and staff use of personal protective equipment in the studied hospitals. The existence of appropriate high-capacity disinfection equipment in hospitals have lead to proper management of increased quantity and potential of infectious in medical waste. Segregation of Covid-19 waste from other medical waste as well as daily disinfection of Covid-19 waste and infectious waste generated in other wards has reduced the possibility of disease transmission from the waste mass, however, the lack of separate containers for disposing of gloves and face masks in hospitals can lead to increase the risk of infection and disease from the medical waste.
